# Targeted mutagenesis in cotton (*Gossypium hirsutum* L.) using the CRISPR/Cas9 system

**DOI:** 10.1038/srep44304

**Published:** 2017-03-13

**Authors:** Xiugui Chen, Xuke Lu, Na Shu, Shuai Wang, Junjuan Wang, Delong Wang, Lixue Guo, Wuwei Ye

**Affiliations:** 1 State Key Laboratory of Cotton Biology/Institute of Cotton Research of Chinese Academy of Agricultural Sciences, Anyang, Henan 455000, China; 2 National Key Laboratory of Crop Genetic Improvement, College of Plant Sciences and Technology, Huazhong Agricultural University, Wuhan, Hubei 430070, China

## Abstract

The CRISPR (Clustered Regularly Interspaced Short Palindromic Repeats)/Cas9 system has been widely used for genome editing in various plants because of its simplicity, high efficiency and design flexibility. However, to our knowledge, there is no report on the application of CRISPR/Cas9-mediated targeted mutagenesis in cotton. Here, we report the genome editing and targeted mutagenesis in upland cotton (*Gossypium hirsutum* L., hereafter cotton) using the CRISPR/Cas9 system. We designed two guide RNAs to target distinct sites of the cotton *Cloroplastos alterados 1 (GhCLA1*) and *vacuolar H*^+^*-pyrophosphatase (GhVP*) genes. Mutations in these two genes were detected in cotton protoplasts. Most of the mutations were nucleotide substitutions, with one nucleotide insertion and one substitution found in *GhCLA1* and one deletion found in *GhVP* in cotton protoplasts. Subsequently, the two vectors were transformed into cotton shoot apexes through *Agrobacterium*-mediated transformation, resulting in efficient target gene editing. Most of the mutations were nucleotide deletions, and the mutation efficiencies were 47.6–81.8% in transgenic cotton plants. Evaluation using restriction-enzyme-PCR assay and sequence analysis detected no off-target mutations. Our results indicated that the CRISPR/Cas9 system was an efficient and specific tool for targeted mutagenesis of the cotton genome.

Genome editing of crop plants is essential for basic and applied research and is thus important for agricultural production. Recently, a new tool for genome editing, the CRISPR (Clustered Regularly Interspaced Short Palindromic Repeats)/Cas (CRISPR-associated) system, was developed from studies of the defence systems of archaea and bacterial that provide protection against invading plasmids and viruses^1^,^2^,^3^. The CRISPR/Cas9 system comprises a CRISPR-associated Cas9 protein and an engineered single guide RNA (sgRNA) that specifies a targeted nucleic acid sequence. The 3′ end of sgRNA forms a hairpin structure that interacts specifically with the Cas9 protein. The first approximately 20 nt of the sgRNA are used to scan genomic DNA to find the desired target DNA sequence. Once complete hybridization between sgRNA and its target DNA sequence has occurred, there is a change in the conformation of the Cas9 molecule and activation of its two nuclease domains. This results in a double-stranded DNA break (DSB).

When a DNA DSB occurs in eukaryotic cells, there are two repair mechanisms: non-homologous end joining (NHEJ) and homologous recombination (HR)^4^. The repair of DSBs by NHEJ can lead to insertion and/or deletion from one to several nucleotides at the break site. This often results in gene knockout when the mutation occurs in an exon of the target gene^5^,^6^. Such mutations in the promoter region of the gene may alter the regulation of gene expression. However, in the HR pathway, the repair of DSBs using homologous donor DNA as a template gives rise to precise gene segment replacement or insertion^7^.

Due to its simplicity, high efficiency and design flexibility, the CRISPR/Cas9 system is widely used for genome editing in various plants^8^,^9^. To date, the CRISPR/Cas9 system has been successfully applied to efficient genome editing not only in model plants species, but also in crop plants species^8^,^9^. There are also tow reports of the successful application of the CRISPR/Cas9 system in a woody species, *Populus tomentosa*^5^,^10^. The transient expression of the CRISPR/Cas9 system successfully generates genome editing in the protoplasts of soybean^6^ and wheat^11^, while the stable transformation of Cas9 and sgRNA mediated by *Agrobacterium tumefaciens* creates gene mutation lines in the first generation of these species. Moreover, the changes of target sequences in the stable transgenic lines can transmit to the next generations in some species, such as *Arabidopsis*^12^ and rice^13^. Although the CRISPR/Cas9 system has been widely and successfully used in many crop plants, there is no report on its efficacy in upland cotton (*Gossypium hirsutum* L.), hereafter referred to as cotton.

As an important resource of fibre, plant protein and edible oil, cotton has tremendous economic value. With the release of the whole-genome sequence of *G. hirsutum* in 2015^14^, much genomic information now provides promise in the study of cotton gene function and improving its resistance to biotic and abiotic stresses. However, compared to *Arabidopsis* and rice, functional genomics research in cotton is more difficult because of its allopolyploidy, low efficiency of genetic transformation and a limited number of mutants. Currently, the CRISPR/Cas9 system offers a new approach to obtain mutants with genes of interest to study gene function in cotton.

Here, we report the application of CRISPR/Cas9-mediated targeted mutagenesis in cotton. Targeted mutagenesis in the *Cloroplastos alterados 1 (GhCLA1*) and *vacuolar H+-pyrophosphatase (GhVP*) genes was achieved by transient and stable transformation. Our results demonstrate that using the CRISPR/Cas9 system can advance cotton functional genomic research and substantially increase the potential for cotton molecular breeding.

## Results

### Construction of CRISPR/Cas9 vectors for the detection of mutagenesis in cotton and target selection

**Figure 1:**
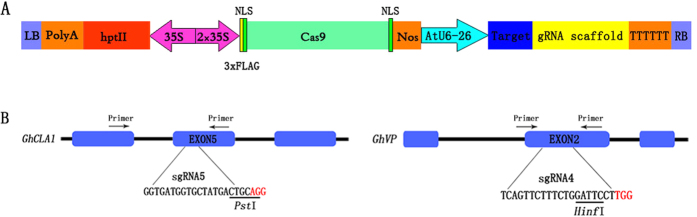
Schematic diagram of Cas9/sgRNA binary vector and target sites selection. (**A**) Schematic illustrating of the Cas9 and sgRNAs expression cassettes in a single binary vector for protoplast transformation and plant stable transformation. NLS, nuclear localization sequence. LB: left border; RB: right border. (**B**) Schematic of target site selection in the two genes with PAM (red). Blue boxes indicate exons; black lines indicate introns. *Pst*I and *Hinf*I sites are underlined.

In our study, to improve co-delivery, both the *Cas9* gene and sgRNA gene were constructed into one expression vector (Fig. 1A). Using the CRISPR/Cas9 system, two endogenous genes of cotton were selected for targeted mutagenesis: *GhCLA1* and *GhVP*. For each gene, a sgRNA gene was designed (Fig. 1B) to contain a restriction site that overlapped with the predicted cleavage site for the Cas9/sgRNA complex – a site located 3 bp upstream of the PAM site. As the cotton cultivar Zhong 9807 is an allotetraploid, we analyzed the sgRNA targets in the two different genomes with specific primers (Supplementary Table S1) and found that the two target sites were the same and the primer sites for PCR amplification of DNA were also identical in the two different genomes (Supplementary Fig. S1). Therefore, there were two binary vectors generated to evaluate targeted mutagenesis in this study.

### Targeted mutagenesis in cotton protoplasts

**Figure 2:**
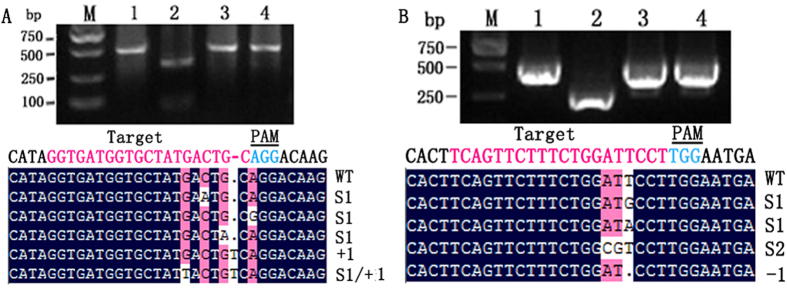
Targeted mutagenesis in cotton protoplasts. (**A**) Detection of mutations at the *GhCLA1* loci. (**B**) Detection of mutations at the *GhVP* loci. Detection of mutations using RE-PCR assay and sequence of the CRISPR/Cas9 system-induced mutation in cotton protoplasts. DNA fragments obtained by PCR amplification of genomic DNA from control protoplasts either undigested (lane 1) or digested (lane 2) with *Pst*I (**A**) or *Hinf*I (**B**). Genomic DNA from transgenic protoplasts were digested before PCR amplification with *Pst*I (**A**) or *Hinf*I (**B**) and, therefore, PCR products were almost exclusively products (lane 4) from mutated DNA sequences. Sequences of the mutations induced by the CRISPR/Cas9 system are shown at the bottom. The change in the number of nucleotides is shown to the right of each sequence. +: insertion; −: deletion; S: substitution.

The activity of the CRISPR/Cas9 system was first verified in cotton protoplasts. The two vectors were transformed into cotton protoplasts, and the genomic DNA of transformed protoplasts was extracted after 48 h of incubation in darkness at room temperature. A restriction enzyme PCR (RE-PCR) assay was used to detect mutations in the targeted genes, as the mutant genes were not digested because of the loss of the enzyme cleavage sites. The PCR results confirmed that the two vectors could induce targeted mutagenesis (Fig. 2). Sequence analysis revealed that most of the mutations were nucleotide substitutions, with one nucleotide insertion and one substitution found in *GhCLA1* and one deletion found in *GhVP* (Fig. 2).

### Targeted mutagenesis in transgenic cotton plants

**Figure 3:**
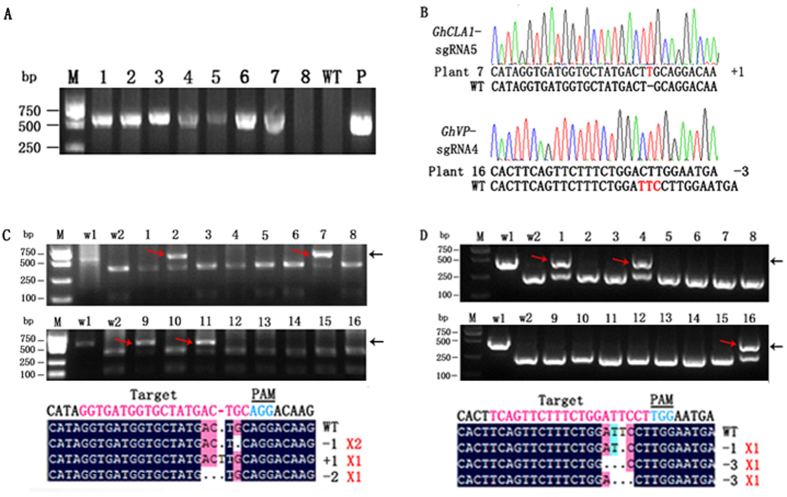
Targeted mutagenesis using the CRISPR/Cas9 system in transgenic cotton plants. (**A**) Identification of *Cas9* gene in transgenic cotton plants. The wild-type DNA and the plasmid of the constructed vector were used as negative (WT) and positive controls (P), respectively. Lanes 1–8 represent different transgenic cotton plants. (**B**) Example chromatograms and representative sequences identified from transgenic plants. Red letters indicate the insertion or deletion of nucleotides. (**C**) Detection of mutations at the *GhCLA1* loci. (**D**) Detection of mutations at the *GhVP* loci. w1 and w2: the undigested and digested PCR products from wild-type DNA, respectively. Lanes 1–16: the digested PCR products of the independent transgenic cotton plants, with mutant DNA are shown with red arrows. The expected fragments were indicated with black arrows. Sequences of the mutations induced by the CRISPR/Cas9 system are shown at the bottom. Red numbers on the right indicate the number of detected transgenic plants with the same mutation type.

To test whether gene mutations detected in cotton protoplasts could also be detected in plants, we verified the presence of the *Cas9* gene in transgenic plants; 22 lines for the *GhCLA1*-sgRNA5 target and 18 lines for the *GhVP*-sgRNA4 target were obtained (Fig. 3A). Then, we identified the target gene mutations in these transgenic lines by RE-PCR assay. PCR products of these two genes were digested with a specific restriction enzyme (Fig. 1B), and undigested bands were observed (Fig. 3C,D). The undigested bands from the RE-PCR assay were cloned and sequenced to confirm the mutations. Sequence analysis indicated that most mutations were nucleotide deletions, while one mutation of *GhCLA1* was a nucleotide insertion (Fig. 3C,D). The mutation rates of these two genes were 47.6–81.8% (Table 1) by directly sequencing the PCR products of the target genes in each transgenic plants. However, we did not find biallelic mutants in transgenic cotton plants according to the RE-PCR assay (Fig. 3C,D) and sequencing analysis (Table 1).

### Off-target analysis in cotton

Previous research showed that the CRISPR/Cas9 system can tolerate several mismatches between the sgRNA gene and its target^15^, so we analyzed the potential off-targets of *GhCLA1*-sgRNA5 and *GhVP*-sgRNA4 in transgenic cotton plants. After searching on the CRISPR-P website (containing the *G. raimondii* genome database) and the *G. hirsutum* genome database with the *GhCLA1*-sgRNA5 and *GhVP*-sgRNA4 target sequences, 22 potential off-target sequences of *GhCLA1*-sgRNA5 and 19 sequences of *GhVP*-sgRNA4 with the PAM motif were identified (Table 2). These identified off-target sites were verified by RE-PCR assay and sequence analysis (Supplementary Tables S3, S4). Only plants containing the verified *GhCLA1* or *GhVP* gene mutations were analyzed for off-target mutations. We managed to assay 30 of the identified off-target loci, and none of these potential off-target loci showed evidence of a CRISPR/Cas9 system-induced mutation (Supplementary Fig. S2). These results indicated that the CRISPR/Cas9 system had high specificity for targeted mutagenesis in cotton.

## Discussion

Targeted mutagenesis is an ideal tool for studying gene function in plants. There are three primary targeted mutagenesis technologies: ZFN, TALEN and CRISPR/-Cas9. Compared with ZFNs and TALENs, the CRISPR/Cas9 system is easy to use and has high efficiency in generating target mutagenesis. Cotton is an allotetraploid and its genome is large and complicated, which makes it difficult to obtain materials with a target gene mutation. In this study, we successfully obtained target mutagenesis in cotton using the CRISPR/Cas9 system. Although most of the mutations in cotton protoplasts were nucleotide substitutions, nucleotide deletions/insertions were found in the transgenic cotton plants. And this results were concordant with that of in soybean^6^. The mutation efficiencies in transgenic plants were 47.6–81.8% (Table 1). This high efficiency of target gene mutation suggests that the CRISPR/Cas9 system can be used to study gene function in cotton.

**Figure 4:**
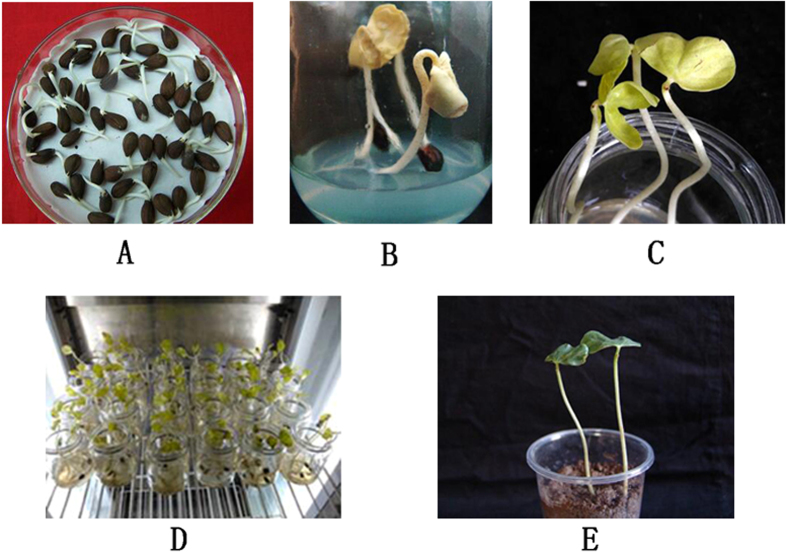
Stable transformation of cotton by using *Agrobacterium* and the shoot apex. (**A** to **E**) represent the process of obtaining stable transgenic cotton plants. (**A**) Sterile cotton seeds were germinated on moistened filter paper in sterile glass plate. (**B**) Seeds with 1–2 cm length root were moved to glass vessels (5 cm in diameter, 8 cm in height) with MS medium (pH 5.8–6.0) and then grew at 28 °C in darkness. (**C**) One of each seedling’s cotyledons was removed and the naked shoot apex was injured with a sterilized scalpel. A small sterile absorbent cotton ball with *Agrobacterium* suspension was placed on the injured shoot apex and vacuum-infiltrated at 0.5 MPa for 10 min at room temperature. (**D**) Infected seedlings were co-cultivated in an incubator at 28 °C for 2 d in darkness and 2 d under light. (**E**) Seedlings were transferred to soil and grew in the greenhouse.

The transformation of the CRISPR/Cas9 system into the cotton genome is an important step for research dealing with gene function and crop improvement. There are three major methods for cotton transformation: the pollen tube pathway-mediated method, the biolistic particle delivery system and *Agrobacterium*-mediated genetic transformation. Among them, *Agrobacterium*-mediated genetic transformation is the most widely used in cotton genetic transformation^16^. In our study, we delivered the CRISPR/Cas9 system into the cotton genome using the *Agrobacterium*-mediated method with the shoot apexes as receptors (Fig. 4). Unlike stem apexes as the explant, the injured shoot apexes can continue to grow on their original plants. Then, the transgenic lines can be tested with genomic DNA from the true leaves growing from the shoot apexes after 3–4 weeks. Although this method is time-saving, it is difficult to obtain homozygotes in the first generation (T_1_). This may be why we did not detected biallelic mutations in the transgenic cotton in the first generation with the CRISPR/Cas9 system. However, as mutations can be stably inherited in the T_2_ and T_3_ generations^12^,^17^, it has great potential to produce biallelic mutations of target genes in the next generation.

The off-target activity of the CRISPR/Cas9 system has been reported in some plant species^8^,^18^. In this study, we also searched for off-target gene mutations for *GhCLA1*-sgRNA5 and *GhVP*-sgRNA4. Searching on the websites CRISPR-P and the cotton genome database with the sgRNA sequence showed 41 candidate sequences with high similarity to *GhCLA1*-sgRNA5 and *GhVP*-sgRNA4 (Supplementary Tables S3, S4). However, we did not detect any mutations in 30 of 64 loci using the RE-PCR assay and sequence analysis. This was not a systematic assessment of the specificity for targeted genome editing using the CRISPR/Cas9 system in cotton, and more candidate off-target sites should be analyzed to evaluate the off-target effect in the future.

In this study, we demonstrated that targeted mutagenesis with the CRISPR/Cas9 system occurred in cotton. Two target genes were edited specifically by the CRISPR/Cas9 system in cotton protoplasts and transgenic plants. Our results indicate that the CRISPR/Cas9 system can not only be used to investigate gene function in cotton, but also has great promise to construct a mutant library for cotton.

## Materials and Methods

### Vector construction

The CRISPR/Cas9 plant gene knockout vector kit (Genloci, Nanjing, China) was used to construct the plant expression vectors. The plasmid of this kit contained a human codon-optimized *Cas9* gene (under the CaMV35 promoter) with a nuclear localization signal (NLS) and a sgRNA gene (under the control of the *Arabidopsis AtU6* gene promoter) (Fig. 1A). The sgRNA scaffold clones with 20-nt target sequences were obtained by PCR using a pair of synthetic specific primers (Supplementary Table S2) according to the manufacturer’s instructions. They were then recombined with the pP1C.4 plasmid that was digested completely with *EcoR*I and *Xba*I to form the Cas9-AtU6-sgRNA vectors specific for different target genes.

### Preparation and transformation of cotton protoplasts

In preparation for generating protoplasts, cotton seedlings were germinated under a 14-/10-h light/dark cycle at 28 °C in a low-humidity chamber. Cotton protoplasts were prepared from fresh etiolated cotyledons of 12-day-old seedlings as described by Yoo *et al*.^19^ with some modifications. Briefly, 6 fresh cotyledons were cut into 0.5-mm strips and immediately transferred into 10 mL of enzyme solution (2.0% cellulase R10, 0.4% macerozyme R10, 0.6 M mannitol, 10 mM MES at pH 5.7, 20 mM KCl, 10 mM CaCl2 and 0.1% BSA) to digest at 25 °C and 50 rpm for 20 h in darkness. The following steps were performed as described by Yoo *et al*.^19^. The protoplasts were re-suspended in MMG solution (15 mM MgCl2, 4 mM MES at pH 5.7, 0.4 M mannitol) for plasmid transformation. The protoplast transformation procedure was performed as described by Yoo *et al*.^19^.

### *Agrobacterium*-mediated transformation of cotton

The stable transformation of cotton was performed using the protocol of Lv *et al*.^20^,^21^,^22^ (Fig. 4). Briefly, seeds of cotton cultivar Zhong 9807 were surface sterilized and germinated on moistened filter paper in glass plate at 28 °C in darkness. When the length of the seed root was 1–2 cm, we moved the seeds into sterilized glass vessels (5 cm in diameter, 8 cm in height) with MS medium. After incubation in an incubator at 28 °C in darkness for 5 d, one of each seedling’s cotyledons was removed and the naked shoot apex was injured with a sterilized scalpel. Then, a small sterile absorbent cotton ball with *Agrobacterium tumefaciens* suspension was placed on the injured shoot apex and vacuum-infiltrated at 0.5 MPa for 10 min at room temperature. Afterward, the infected seedlings were co-cultivated for 2 d in darkness and 2 d under light and then transferred to the greenhouse. Transgenic plants were selected with 2.5 g/L kanamycin at the three-leaf stage.

### Detection of mutations

Genomic DNA was isolated by a DNAquick Plant System (Tiangen, Beijing, China) according to the manufacturer’s protocol. To detect mutations in cotton protoplasts, the genomic DNA was digested with restriction enzymes (*Pst*I for *GhCLA1*-sgRNA5 and *Hinf*I for *GhVP*-sgRNA4). After digestion, the target genes were amplified with gene-specific primers (Supplementary Table S1), and the PCR fragments were ligated to a pLB vector using a Lethal Based Fast Cloning Kit (Tiangen) for sequencing. The PCR contained 1 × PCR buffer (include MgCl_2_), 200 μM dNTP, 1.25 U PrimeSTAR GXL DNA Polymerase (TaKaRa, Dalian, China), 100 ng template and 0.2 μM of each primer, and was subjected to a regime of 35 cycles of 98 °C for 10 s, 55 °C for 15 s, and 72 °C for 40 s.

To detect mutations in transgenic cotton lines, the target genes were amplified by PCR using gene-specific primers (Supplementary Table S1). The PCR segments were purified using a Cycle-Pure Kit (Omega, Guangzhou, China) and digested for 4 h with either *Pst*I or *Hinf*I. The digested products were separated by electrophoresis in a 1.5% agarose gel and the undigested bands were purified with an Easy Pure Quick Gel Extraction kit (Tiangen) and then ligated into a pLB vector (Transgen, Beijing, China) for sequencing to detect gene mutations. The mutation rate in transgenic plants was calculated by directly sequencing the PCR products of the target genes in each transgenic plants and according to the ratio of mutated clonal amplicons versus total sequenced clonal amplicons. More than 20 clonal amplicons of each transgenic plants were sequenced.

### Off-target analysis

Potential off-target sites were identified on the websites CRISPR-P (http://cbi.hzau.edu.cn/cgi-bin/CRISPR, which contains the *G. raimondii* genome database) and the *G. hirsutum* reference genome (http://cgp.genomics.org.cn/page/species/index.jsp). First, we searched the potential off-target sites on the CRISPR-P website and chose the top 20 potential off-target sequences. Then, we searched the top 20 sequences in the *G. hirsutum* genome database and selected the potential off-target sites and their loci. Afterward, we identified most of them using the RE-PCR assay and sequencing. The first two potential off-target sites of *GhCLA1*-sgRNA5 were identified by sequencing because there were no available enzyme recognition sequences near the PAM and more than 10 clones were sequenced.

## Additional Information

**How to cite this article:** Chen, X. *et al*. Targeted mutagenesis in cotton (*Gossypium hirsutum* L.) using the CRISPR/Cas9 system. *Sci. Rep.* **7**, 44304; doi: 10.1038/srep44304 (2017).

**Publisher's note:** Springer Nature remains neutral with regard to jurisdictional claims in published maps and institutional affiliations.

## Supplementary Material

Supplementary Information

## Figures and Tables

**Table 1:** Gene mutations in two target genes using the CRISPR-Cas9 system.

Target gene	No. of examined plants	No. of plants with *Cas9* gene	No. of plants with mutation	Mutation rates of transgenic plants (Plant ID/mutation rate)
*GhCLA1*	892	22	4	2/55.0%, 7/81.8%, 9/80.0%, 11/73.7%
*GhVP*	779	18	3	1/55.0%, 4/57.9%, 16/47.6%

**Table 2:** Analysis of mutations in potential off-target sites in transgenic plants.

sgRNA	No. of putative off-target sites	No. of loci of putative off-target sites	No. of examined loci	No. of plants examined	No. of off-target sites
*GhCLA1*-sgRNA5	22	36	13	4	0
*GhVP*-sgRNA4	19	28	17	3	0

Only plants containing the verified *GhCLA1* or *GhVP* gene mutations were analyzed for off-target mutations.
