# Supplementary material for: Targeted mutagenesis in cotton (Gossypium hirsutum L.) using the CRISPR/Cas9 system
Source: Sci Rep. 2017 Mar 13;7:44304. doi: 10.1038/srep44304 (PMC5347080; doi:10.1038/srep44304)
Supplement: Supplementary Information [file srep44304-s1.pdf]

Supplementary Information

Targeted mutagenesis in cotton (*Gossypium hirsutum* L.) using the CRISPR/Cas9 system

Xiugui Chen[1, 2], Xuke Lu[1], Na Shu[1], Shuai Wang[1], Junjuan Wang[1], Delong Wang[1], Lixue Guo[1] and Wuwei Ye[1,*]

[1] State Key Laboratory of Cotton Biology/Institute of Cotton Research of Chinese Academy of Agricultural Sciences, Anyang, Henan 455000, China

[2] National Key Laboratory of Crop Genetic Improvement, College of Plant Sciences and Technology, Huazhong Agricultural University, Wuhan, Hubei 430070, China

*Author for correspondence. WY: yew158@163.com.

Supplementary Information contains:

Supplementary Figures S1-S2

Supplementary Tables S1-S4

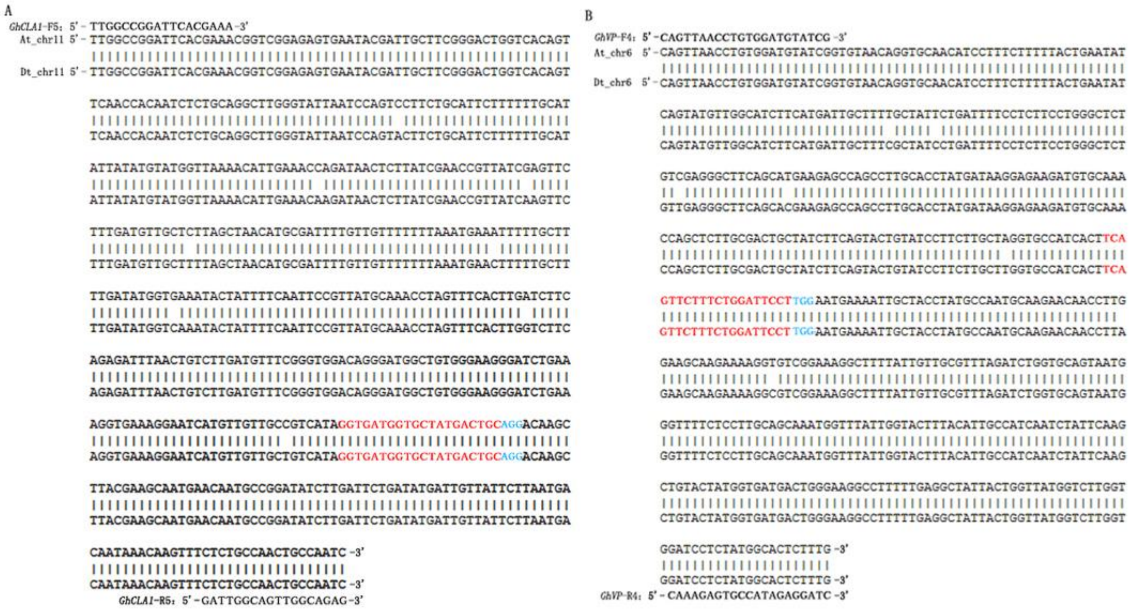

Supplementary Fig. S1. Identification of target sites and the primer sites for PCR amplification of DNA in the two different genomes. (**A**) Sequences of the PCR fragment of *GhCLA1*. (**B**) Sequences of the PCR fragment of *GhVP*. Red letters represent the target sites, blue letters represent the PAM sequence.

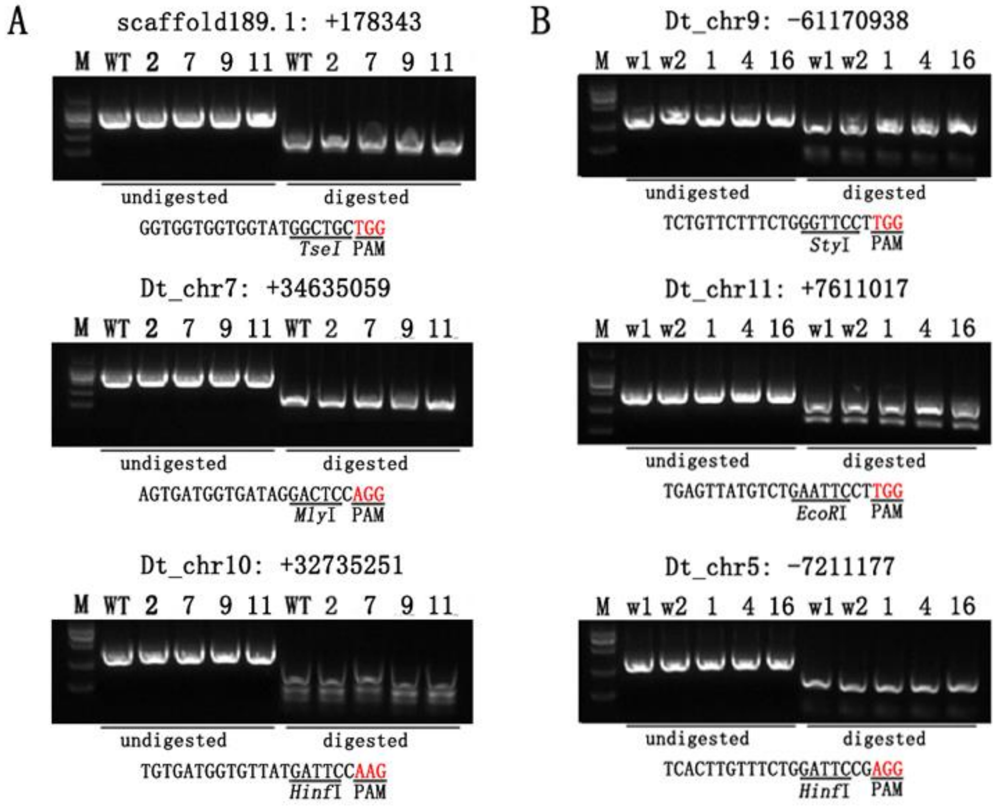

Supplementary Fig. S2. Examples of restriction-site loss assays with potential off-targets. (**A**) Analysis examples of potential off-targets from plants containing verified *GhCLA1* gene mutations. (**B**) Analysis examples of potential off-targets from plants containing verified *GhVP* gene mutations. The restriction enzyme sites are showed with an underscore. The PAMs are highlighted with red.

Supplementary Table S1. List of primers in this study.

Usage	Primer name	Sequence (5'-3')
Cloning	*Cas9*-F	TCCTGCAGACAGTGAAGGTG
	Cas9-R	GCCTTATCCAGTTCGCTCAG
Mutation detection	*GhCLA1*-F5	TTGGCCGGATTCACGAAA
	GhCLA1-R5	GATTGGCAGTTGGCAGAG
	GhVP-F4	CAGTTAACCTGTGGATGTATCG
	GhVP-R4	CAAAGAGTGCCATAGAGGATC

Supplementary Table S2. Target sequences of two genes in cotton and oligonucleotides used to express sgRNA in the vectors.

Target gene	Target site sequences	Oligo forward (5'-3')	Oligo reverse (5'-3')	Enzyme
GhCLA1	GGTGATGGTGCTATGACTGCAGG	CAGGAAACAGCTATGACCATATTCATTCGGAGTTTTTGTATC	gctatttctagctctaaaacGCAGTCATAGCACCATCACCaatcacacttcgactct	*Pst*I
GhVP	TCAGTTCTTTCTGGATTCCTTGG	CAGGAAACAGCTATGACCATATTCATTCGGAGTTTTTGTATC	gctatttctagctctaaaacAGGAATCCAGAAAGAACTGACaatcactacttcgactct	*Hinf*I

The restriction enzyme sites are showed with an underscore. The PAMs are highlighted with red.

Supplementary Table S3. List of potential off-target sites identified for the *GhCLA1* target sequence in the *G. hirsutum* genome.

Sequence of the putative off-target site	Putative off-target locus	Number of matching bases (include PAM)	Available restriction enzyme cutting site	Tested?
GGAGATGGAGCTATGACTGCTGG	At_chr10: -32691215	20	NO	YES
	Dt_chr8: -13606802	20	NO	YES
GGTGATGGAGCAATGACTGCTGG	Dt_chr13: +22584502	20	NO	YES
	Dt_chr13: +22620167	20	NO	YES
	scaffold72.1: -1097120	20	NO	YES
	scaffold72.1: -1130168	20	NO	NO
	scaffold72.1: -1164191	20	NO	NO
GGTGGTGGTGGTATGGCTGCTGG	scaffold189.1: +178343	19	TseI	YES
	At_chr9: -4372202	19	TseI	YES
AGTGATGGTGATAGGACTCCAGG	scaffold2376.1: -159740	19	MlyI	YES
	Dt_chr7: +34635059	19	MlyI	YES
GGTGATGGTGCTGGGCCTGCTGG	Dt_chr13: +16377109	19	Cac8I	YES
GGTGGTGGTGAAATGACTGGGGG	Dt_chr7: +14078964	18	BsrI	YES
TGTGATGGTGTTATGATTCCAAG	Dt_chr10: +32735251	18	HinfI	YES
GGTGATGGTGGTGGCACTGCTGG	At_chr8: -16619447	18	BtsI	YES
GGTGATGGTGGTGGCAATGCTGG	Dt_chr13: -46985487	17	BsrDI	NO
GGTGATGGTGGGATGACACCAGG	Dt_chr13: +20112017	19	NO	NO
	Dt_chr7: -19747713	19	NO	NO
GGTTATGGTGTCATGATTGCAGG	Dt_chr13: -15976702	19	NO	NO
GGTGATGGAGCAATGACGGCTGG	Dt_chr13: +22563464	19	NO	NO
GATGATGGTGCTAGGACTACAAG	Dt_chr4: -32253836	19	NO	NO
GGTGACTGTGCTAAGCCTGCTGG	Dt_chr7: -35412683	18	NO	NO
AGTGAGGTTGCTATGAGTGCAAG	Dt_chr1: -63423097	18	NO	NO
GGTGAGGTTGCTGTGGCTGCTGG	scaffold3130.1: +69411	18	NO	NO
GGTTATGGTACTATGAGTGTAAG	Dt_chr5: -59686925	18	NO	NO
GGTTATGGCACTATGAGTGTAAG	Dt_chr1: +11783798	18	NO	NO
	Dt_chr1: -80500846	18	NO	NO

The restriction sites are shown with an underscore; mismatching bases are shown in blue.

Supplementary Table S3. List of potential off-target sites identified for the *GhCLA1* target sequence in the *G. hirsutum* genome (continued).

Sequence of the putative off-target site	Putative off-target locus	Number of matching bases (include PAM)	Available restriction enzyme cutting site	Tested?
GGTGACTGTGCTGAGCCTGCTGG	Dt_chr1: +65502164	17	NO	NO
	At_chr2: +58074573	17	NO	NO
	Dt_chr11: +25261498	17	NO	NO
	Dt_chr11: +54287243	17	NO	NO
	Dt_chr10: -39718103	17	NO	NO
	Dt_chr4: +25237184	17	NO	NO
GGTGACTATGCTAAGCCTGCTGG	At_chr2: +58103544	17	NO	NO
GTTGCTTATGCTATGACTGATGG	Dt_chr5: +1180457	17	NO	NO
GTTGATGGTGCCATAAGTGGTGG	Dt_chr13: +21995540	17	NO	NO

The restriction sites are shown with an underscore; mismatching bases are shown in blue.

Supplementary Table S4. List of potential off-target sites identified for the *GhVP* target sequence in the *G. hirsutum* genome.

Sequence of the putative off-target site	Putative off-target locus	Number of matching bases (include PAM)	Available restriction enzyme cutting site	Tested?
TCTGTTCTTTCTGGGTTCCTTGG	Dt_chr9: -61170938	21	StyI	YES
TCAGTTCTTATTGGATTGCTTGG	Dt_chr4: +27630888	20	Tth111II	YES
	Dt_chr4: +27631776	20	Tth111II	YES
	At_chr4: +87726589	20	Tth111II	YES
	At_chr4: +87727477	20	Tth111II	YES
	At_chr4: +87728332	20	Tth111II	NO
TCAGGTCTTTGAGGATTCCTTGG	Dt_chr9: +42031687	20	HinfI	YES
TGAGTTATGTCTGAATTCCTTGG	Dt_chr11: +7611017	19	EcoRI	YES
	At_chr4: +71425367	19	EcoRI	YES
TGAGTTATATCTGAATTCCTTGG	At_chr11: -60320285	19	EcoRI	YES
TCACTTGTTTCTGGATTCCGAGG	Dt_chr5: -7211177	19	HinfI	YES
TCTGTTGTTACTGGATTCCTTAG	At_chr6: +53150767	19	HinfI	YES
TCAGATCATTCTTGATTCCACGG	At_chr12: -22967324	18	HinfI	YES
	At_chr3: +27499491	18	HinfI	YES
TCTGTTGTAACTGGATTCCTTAG	Dt_chr6: +11310880	18	HinfI	YES
TCAGATCATTCTTGATTCCGCGG	Dt_chr9: -42381100	18	HinfI	YES
TCCTTTCTTTGTGGATGCCTCGG	scaffold165.1: +486549	18	SfaNI	YES
TTAGTAGTTTCAGGATTCCTTAG	Dt_chr11: -41707429	18	HinfI	YES
TCATTTCTTCGTAGATGCCTCGG	At_chr2: -22997961	17	SfaNI	NO
TCATTTCTTCGCGGATGCCTCGG	At_chr9: -47177313	17	SfaNI	NO
TCCTTTCTTTGTAGATGCCTCGG	At_chr3: -41473876	17	SfaNI	NO
	Dt_chr1: +10912255	17	SfaNI	NO
	At_chr9: +49349713	17	SfaNI	NO
TCAGATCTTTCTTGTTTCCTAGG	scaffold4724.1: -30530	19	NO	NO
TCATTTCTTTTTGGATTTTTAGG	Dt_chr8: +15208796	18	NO	NO
TAATTTGTTTCTGGGTTCCTTAG	Dt_chr11: +12832761	18	NO	NO
TAATTTGTTTCTGGGTTCTTTAG	Dt_chr2: +13309509	17	NO	NO
	At_chr2: +60047189	17	NO	NO

The restriction sites are shown with an underscore; mismatching bases are shown in blue.
